# Identification and Expression Characterization of ATP-Binding Cassette (ABC) Transporter Genes in Melon Fly

**DOI:** 10.3390/insects12030270

**Published:** 2021-03-23

**Authors:** Hui-Qian Xu, Meng Ma, Yun-Peng Ma, Su-Yun Zhang, Wei-Jun Li, Dong Wei, Jin-Jun Wang

**Affiliations:** 1 Chongqing Key Laboratory of Entomology and Pest Control Engineering, College of Plant Protection, Southwest University, Chongqing 400715, China; endeavorx@163.com (H.-Q.X.); mameng202101@163.com (M.M.); mypeng159@163.com (Y.-P.M.); 18375633196@163.com (S.-Y.Z.); liweijun201608@163.com (W.-J.L.); 2 State Cultivation Base of Crop Stress Biology for Southern Mountainous Land, Academy of Agricultural Sciences, Southwest University, Chongqing 400715, China

**Keywords:** *Zeugodacus cucurbitae*, ABC transporter, transcriptional expression, insecticides, detoxification

## Abstract

**Simple Summary:**

The melon fly, *Zeugodacus cucurbitae*, is an important agricultural pest. At present, chemical pesticide treatment is the main method for field control, but this promotes pesticide resistance by *Z. cucurbitae*, because of its frequent use. ABC transporters are involved in detoxification metabolism, but few studies have yet considered their expression in melon fly. In this study, we identified the ABC transporters genes at a genome-wide level in melon fly, and analysed their spatiotemporal expression patterns, as well as changes in expression after insecticides treatments. A total of 49 ABC transporters were identified, and their expression levels varied at different developmental stages and between tissues. After three insecticides treatment, *ZcABCB7* and *ZcABCC2* were up-regulated. After β-cypermethrin induction, tissues were dissected at 12, 24 and 48 h, and the expression levels of a number of ABC genes were highly expressed within the fat body. From these results, we conclude that *ZcABCB7* and *ZcABCC2* may be involved in detoxification metabolism, and that the fat body is the main tissue that plays this role.

**Abstract:**

The ATP-binding cassette (ABC) transporter is a protein superfamily that transports specific substrate molecules across lipid membranes in all living species. In insects, ABC transporter is one of the major transmembrane protein families involved in the development of xenobiotic resistance. Here, we report 49 ABC transporter genes divided into eight subfamilies (ABCA-ABCH), including seven *ABCAs*, seven *ABCBs*, 10 *ABCCs*, two *ABCDs*, one *ABCE*, three *ABCFs*, 16 *ABCGs*, and three *ABCHs* according to phylogenetic analysis in *Zeugodacus cucurbitae*, a highly destructive insect pest of cucurbitaceous and other related crops. The expressions level of 49 ABC transporters throughout various developmental stages and within different tissues were evaluated by quantitative transcriptomic analysis, and their expressions in response to three different insecticides were evaluated by quantitative real-time polymerase chain reaction (qRT-PCR). These ABC transporter genes were widely expressed at developmental stages but most highly expressed in tissues of the midgut, fat body and Malpighian tube. When challenged by exposure to three insecticides, abamectin, β-cypermethrin, and dinotefuran, the expressions of *ZcABCB7* and *ZcABCC2* were significantly up-regulated. *ZcABCB1*, *ZcABCB6*, *ZcABCB7*, *ZcABCC2*, *ZcABCC3*, *ZcABCC4*, *ZcABCC5,* and *ZcABCC7* were significantly up-regulated in the fat body at 24 h after β-cypermethrin exposure. These data suggest that *ZcABCB7* and *ZcABCC2* might play key roles in xenobiotic metabolism in *Z. cucurbitae*. Collectively, these data provide a foundation for further analysis of ABCs in *Z. cucurbitae*.

## 1. Introduction

ATP-binding cassette (ABC) proteins comprise one of the largest superfamilies of prokaryote and eukaryote membrane proteins [1]. According to sequence homology and structural similarity, they can be divided into different subfamily members. For example, a total of 48 ABC transporters in humans are divided into seven subfamilies (ABCA to ABCG) [2], while in *Drosophila melanogaster*, an eighth subfamily (ABCH) was first discovered. This latter member is widely expressed in arthropods and zebrafish but not in mammals, plants and fungi [3,4]. The majority of these ABC proteins comprise two highly conserved structural domains, a nucleotide-binding domain (NBD) that can bind and hydrolyze ATP, and a transmembrane domain (TMD) that is comprised of six membrane-spanning helices [5]. The highly conservative NBD contains three motifs: a Walker A and Walker B domain and the ABC signature motif (LSSG-motif). There are two of these structural domains in ABC transporters from eukaryotes. These are either half-transporters which have one TMD and one NBD, or full-transporters which have two TMDs and two NBDs, and often form a functional unit as a homo- or heterodimer [1,6]. Up until now, the ABC transporters have been widely identified at a genome-wide level in many insect species.

In terms of different functions, ABC transporters can be divided into three categories: input proteins, output proteins and non-transport proteins [7]. The main function of the input and output proteins is substrate transport, e.g., amino acids, lipids, lipopolysaccharides, peptides, inorganic ions, drugs, metals and xenobiotics [8]. Non-transport proteins have no transport function, but are involved in intracellular DNA repair, transcription and regulation of gene expression [9]. In insects, studies have shown that ABC transporters have roles in development and detoxification, including the metabolism of pesticides, plant secondary metabolic compounds (allelochemicals) and other heterologous substances [10]. In recent years, the important role of ABC transporters in the insect’s response to pesticides and other toxic compounds has caused widespread concern [11,12]. In *Bemisia tabaci*, the ABCG3 subfamily may contribute to imidacloprid detoxification [13]. In *Plutella xylostella*, four ABC subfamilies (ABCA, ABCC, ABCG, and ABCH) were increased in a fipronil resistant strain [14]. Similarly, after insecticide exposure, some ABC transporters were up-regulated in mosquito larvae [15]. In *Aphis gossypii*, ABC transporters are involved in thiamethoxam resistance [16]. In addition, inhibition of ABC transporter gene expression by RNA interference (RNAi) can increase pesticide toxicity [17,18].

Although the contribution of ABC transporters to drug resistance in mammals has been extensively studied, the mechanisms by which these ABC transporter genes confer resistance in arthropods remains unclear. The ABCB subfamily contains multi-drug resistance proteins (MDRs) or P-glycoproteins (P-gps). The ABCC subfamily includes the multidrug-resistance associated proteins (MRPs) in humans, and these are involved in the multidrug resistance of cancer cells to chemotherapy [4]. The detoxification processes in insects are divided into three phases: cytochromes P450 monooxygenase (P450s) and carboxylesterases (CarEs) belong to phase I metabolism, glutathione *S*-transferases (GSTs) belongs to phase II, while ABC transporters belongs to phase III. Phases I and II can act directly upon a toxin molecule, and then the substrates transported extracellular in phase III [19,20]. Therefore, it is crucial to understand the role of ABC for conferring resistance to insecticides.

The melon fly *Zeugodacus cucurbitae* (Coquillett) (Diptera, Tephritidae) is an important, global, agricultural pest [21]. *Z. cucurbitae* can infest more than 130 host plants including vegetables and fruits, but mainly gourd and nightshade plants, such as cucumber, pumpkin, melon, watermelon, bitter melon, tomato and eggplant [22] Although the biological control by releasing of parasitoids against *Z. cucurbitae* was evaluated recently [23], the extensive use of pesticides is the frequently method to control *Z. cucurbitae*, which leads to serious resistance to a range of insecticides. At present, there are few studies that have considered the ABC transporters present within the melon fly. Moreover, whether ABC proteins are also involved in different insecticide detoxifications has not been investigated in *Z. cucurbitae*. In this study, we identified 49 ABC transporters from *Z. cucurbitae*. They were divided into eight subfamilies of ABCA~ABCH. The gene expression profiles were studied at different development stages and within various tissues. Secondly, we focused on an analysis of the expression of ABC transporters after three different insecticide treatments. Collectively, a more comprehensive analysis of ABC genes will provide an insight into the physiological function of ABC transporters, and how this is regulated in response to xenobiotic stimuli.

## 2. Materials and Methods

### 2.1. Insects

*Z. cucurbitae* were initially collected from Haikou of Hainan Province, China, in 2016, and maintained in the laboratory under conditions of 27 ± 0.5 °C, 14-h L: 10-h D photoperiod, 70 ± 5% relative humidity (RH). The insects were fed on an artificial diet, as previously described [24].

### 2.2. Identification, Characterization and Phylogenetic Analysis

To identify ABC transporter genes, we carried out tBlastn searching against the *Z. cucurbitae* genome [25], with highly conserved NBDs present in *D. melanogaster* ABC transporter genes. The putative ABC gene sequences were taken as the query objects, and each of the ABC transporter genes was confirmed in the NCBI protein database.

The conserved domains of these *Z. cucurbitae* ABC proteins were predicted using SMART (http://smart.embl.de, accessed on 14 May 2020) [26]. The signal peptide was analyzed with the online software, SignalP 5.0 Server (http://www.cbs.dtu.dk/services/SignalP, accessed on 14 May 2020). Subcellular localization was predicted using TargetP 2.0 [27]. Transmembrane domains were predicted with the software TMHMM Server v2.0 (http://www.cbs.dtu.dk/services/TM-HMM, accessed on 14 May 2020). The N-glycosylation and O-glycosylation sites were analyzed by NetNGlyc1.0 Server (http://www.cbs.dtu.dk/services/NetNGlyc, accessed on 2 July 2020) and NetOGlyc 4.0 server (http://www.cbs.dtu.dk/services/NetOGlyc, accessed on 2 July 2020) [28]. Full-length protein sequences were utilized to construct the respective phylogeny trees using the neighbor-joining method (1000 replicates for bootstrapping) via MEGA 7.0 software.

### 2.3. RNA Isolation and cDNA Synthesis

Three tissues (midgut, fat body, and Malpighian tubule) were dissected from 20 female adult flies, with four replicates of each that includes 4-5 individuals. TRIzol reagent (Invitrogen, Carlsbad, CA, USA) was used to extract total RNA according to the manufacturer’s instructions. The concentration and purity of 1.5 μL of dissolved RNA for each of the samples were quantified using a Nanodrop ONE spectrophotometer (Thermo Fisher Scientific, Waltham, MA, USA) at absorbance ratios of OD 260/280 and OD 260/230. The integrity of RNA was further confirmed after resolution by 1.0% agarose gel electrophoresis. Synthesis of cDNA was performed using the PrimeScript RT Reagent Kit with gDNA Eraser (Takara, Dalian, China) to remove genomic DNA, following the manufacturer’s protocol.

### 2.4. Expression Profiles

Expression profiling of ABC transporters was evaluated using transcriptome data. To determine the expression patterns of ABC transporter genes, the midgut, fat body, Malpighian tubule, ovary and testis of male and female, respectively, were harvested at the following developmental stages: egg; 1-day, 3-day, and 7-day-old larva; 1-day, 5-day, and 9-day-old pupa, as well as 1-day, 5-day, and 9-day-old male or female adults. Total RNA and cDNA preparation was performed as described above. The real-time quantitative PCR (RT-qPCR) was run on a CFX384 Real-time System (Bio-Rad, Singapore) using the NovoStar SYBR qPCR SuperMix (Novoprotein Scientific, Shanghai, China). The qRT-PCR program was run with the following conditions: 95 °C for 2 min, 40 cycles of 95 °C for 15 s, 60 °C for 30 s, and 72 °C for 30 s. A melting curve analysis at 60–95 °C was performed to ensure the specificity of each primer. In addition, *α-tubulin* and *β-tubulin 1* [29] were used as reference genes for normalization. The primers used for ABC transporters validation were designed using Primer 3.0 (http://bioinfo.ut.ee/primer3-0.4.0/) (Table S1). All reactions were undertaken with two technical replicates and three biological replicates. The relative expression level of the ABC genes was calculated using qBase [30].

### 2.5. Insecticide Exposures

The transcriptional responses of certain ABC genes of *Z. cucurbitae* to three commonly used insecticides were investigated, including one neonicotinoid, dinotefuran (98% purity, Hailir, Shandong, China), one biogenic pesticide, abamectin (95% purity, Bangnong, Guangdong, China), and one pyrethroid, β-cypermethrin (93% purity, LIER-Chemical, Sichuan, China). Insecticides were dissolved in acetone, and then diluted to achieve five or six different concentrations: dinotefuran (at 1000, 700, 500, 250, 100 mg/L), abamectin (at 120, 60, 30, 15, 7.5, 3.75 mg/L) and β-cypermethrin (at 480, 240, 120, 80, 60 mg/L). Newly emerged adults were collected and sexually separated in two cages. Five-day-old virgin female adults were anesthetized by exposure to CO_2_ for 2 min and then treated with insecticide (0.5 μL per fly) by dropping it onto their pronotum. For each concentration, approximately 20 adult flies were placed in a plastic cup. *Z. cucurbitae* were treated with acetone solutions as control. Each concentration of the insecticides was performed with three replicates. After 48 h, the mortality of *Z. cucurbitae* was recorded. If the tested flies did not move after stimulation with a camel hairbrush, they were considered to be dead. The concentration producing either 50% or 30% lethality (LC_50_ and LC_30_, respectively) was calculated using PoloPlus. Three insecticides, dinotefuran (LC_50_, 296.01 mg/L; LC_30_, 219.62 mg/L), abamectin (LC_50_, 14.66 mg/L; LC_30_, 9.34 mg/L) and β-cypermethrin (LC_50_, 125.91 mg/L; LC_30_, 100.58 mg/L) were used in this study. Insecticides were diluted in acetone at the experimentally-determined LC_30_ and LC_50_ concentrations. Using the same method (as above) to treat five-day-old virgin female adults, acetone was used as control. In total, 120 adults were treated with insecticides. Surviving flies after each treatment were randomly collected for RNA extraction at 12 h, 24 h and 48 h post-exposure. Four adults from each time points were collected and RNA extracted. There were four biological replicates for each treatment. *Ribosomal protein subunit 3* and *Ribosomal protein L13* were selected as reference genes evaluated before the quantification (Table S1).

### 2.6. Statistical Analysis

Relative gene expressions within tissues at each developmental stage were analyzed using SPSS 25.0 (SPSS Inc., Chicago, IL, USA). A *p* < 0.05 was regarded as statistically significant.

## 3. Results

### 3.1. Identification and Phylogenetic Analysis of ABC Transporter Genes

Based upon the genome sequence, we identified 49 putative ABC genes in *Z. cucurbitae*. These genes were divided into eight subfamilies: seven *ABCAs*, seven *ABCBs*, 10 *ABCCs*, two *ABCDs*, one *ABCEs*, three *ABCFs*, 16 *ABCGs*, and three *ABCHs* (Table 1), according to the highly conserved NBDs of *D. melanogaster* ABC transporter genes. All of the putative genes had complete ORFs. The 49 ABC transporters were retrieved using the NCBI protein database with its known homologous sequences. The NBD and TMD were further determined using the Pfam program to determine the 49 ABC transporters. Each potential *ZcABCs* is classified into two possible structural forms, full transporters (FTs) or half transporters (HFs). All of the ABCA subfamily belongs to FTs, *ZcABCD*, *ZcABCG*, and *ZcABCH* belong to HFs, and *ZcABCG13* belongs to the FTs family. While some of the genes such as *ZcABCB* and *ZcACC* belong to FTs and the others belong to HFs. *ZcABCA*, *ZcABCB*, *ZcABCC* and *ZcABCD* subfamilies contained both TMD and NBD organization, but *ZcABCG* and *ZcABCH* subfamilies formed NBD-TMD organization. However, no TMD motif was predicted for the *ZcABCEs* and *ZcABCFs* subfamilies.

**Figure 1:**
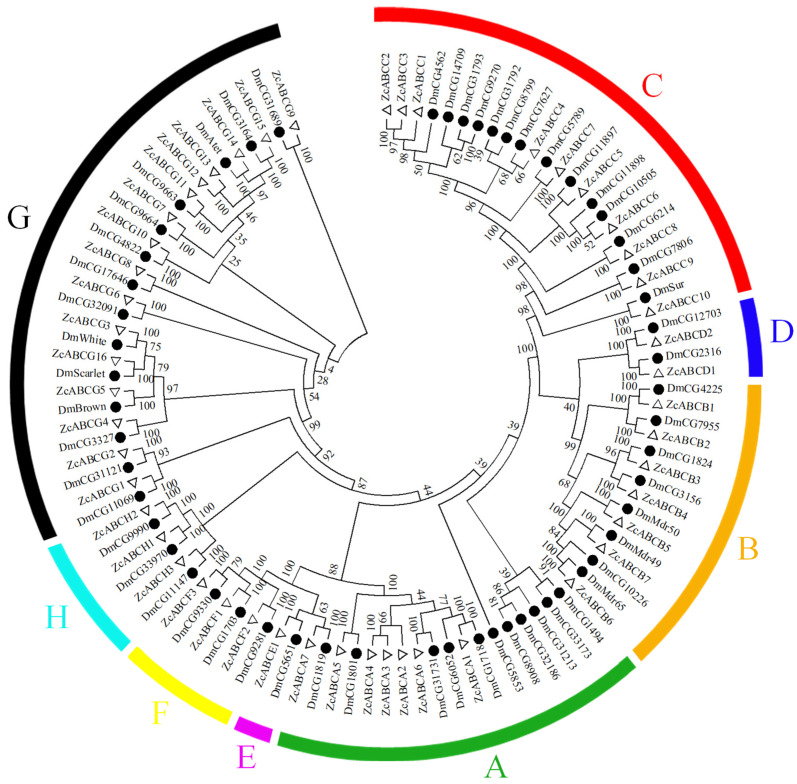
Phylogenetic analysis of ABC transporters in *Zeugodacus cucurbitae*. The phylogenetic tree was constructed by ClustalW alignment using the nucleotide binding domains (NBDs) of ABC transporters from *Z. cucurbitae* and *Drosophila melanogaster*. The number at the branch point of the bootstrap level was from 1000 replications with MEGA 7.0 using the maximum-likelihood method. The hollow diamond and solid circle represent different ABC proteins from *Z. cucurbitae* and *D. melanogaster*, respectively. Capital letters **A**–**H** with different colors indicate the different subfamilies of ABC transporters.

To determine the homology and phylogenetic relationships, 49 ABC transporter proteins were derived from the NCBI Nt database. A phylogenetic tree was constructed based upon presence of ZcABC transporter proteins and DmABC transporter proteins using NDBs domains. Phylogenetic analysis indicated that melon fly ABC transporters can be divided into eight subfamilies (listed A-H) (Figure 1). Phylogenetic analysis revealed that *ZcABCs* were highly similar to *DmABCs*, with the same subfamily clustered on the same branches.

### 3.2. The Expression Pattern of ZcABCs

**Figure 2:**
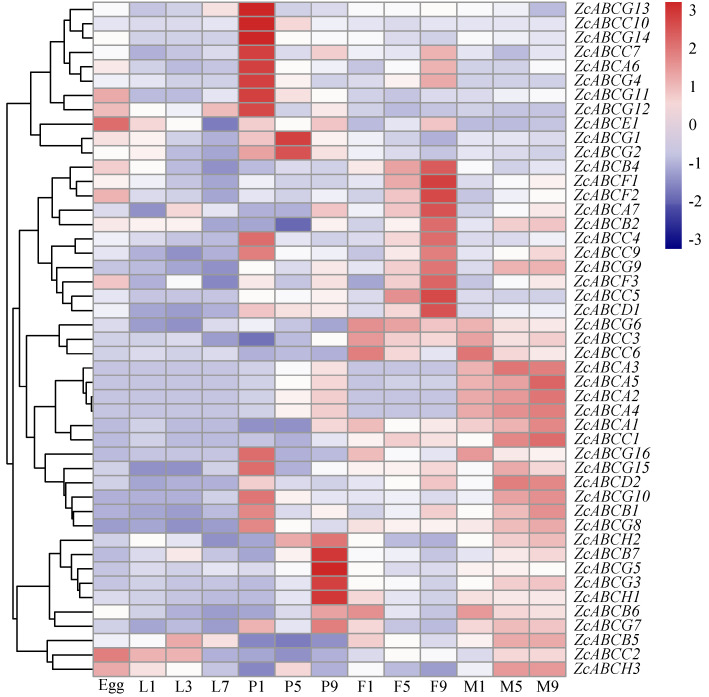
Expression profiles of ABC transporters at different development stages of *Zeugodacus cucurbitae*. Letters on the right are the gene names. The mRNA levels, represented by normalized log_2_ (FPKM + 1) values, are shown in the gradient heat map with colors ranging from blue (low expression) to red (high expression). L1, 1-day-old larva, L3, 3-day-old larva, L7, 7-day-old larva. In the same way, P represents pupa, F represents female adult and M represents male adult, the number represents the day.

The expressions of 49 ABC transporter genes across developmental stages and tissues in *Z. cucurbitae* adults with RPKM > 0 were analyzed. Regarding developmental stages, the majority of *ZcABCs* were expressed at low levels in egg and larva stages. We detected multiple *Z. cucurbitae* ABC genes that are highly expressed during the pupation and adult stage. The analysis revealed that the expression of eight ABC genes and nine ABC genes were expressed in increasing amounts as the female adult and the male adult developed, respectively. Some ABC genes are highly expressed in one day pupa (Figure 2).

**Figure 3:**
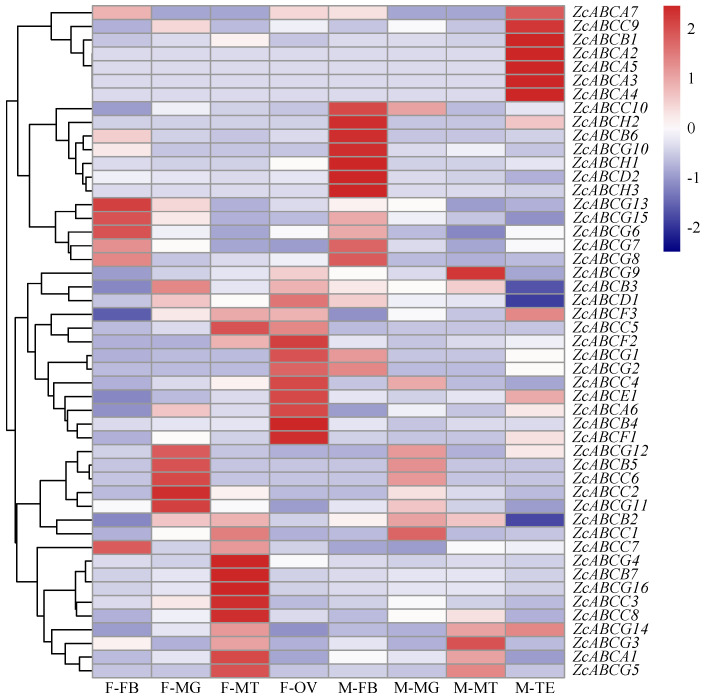
Expression profiles of ABC transporter genes in different tissues of *Zeugodacus cucurbitae*. The relative expression of each gene was normalized using log_2_ (FPKM + 1), and shown in the gradient heat map with colors. FB, fat body, MG, Malpighian tubule, MT, midgut, OV, ovary, and TE, testis. F represents female adult and M represents male adult.

Moreover, we evaluated the levels of 49 *ZcABCs* transporters genes in different tissues. More than half of the *Z. cucurbitae* ABC genes were expressed in Malpighian tubule and midgut (Figure 3). Some tissue-specific ABC genes were identified, e.g., *ZcABCA2*, *ZcABCA3*, *ZcABCA4,* and *ZcABCA5* in the testis, and *ZcABCA6*, *ZcABCB4*, *ZcABCE1*, *ZcABCF2* in the ovary. Some of these genes corresponded to the high expression at adult stage.

### 3.3. Transcriptional Responses of ZcABCs to Insecticides Exposures

In order to evaluate the relative expression of *ZcABCBs* and *ZcABCCs* in response to insecticides exposures, five-day-old female adults were firstly treated with different insecticides and the levels of mortality quantified. The LC_50_ values of the insecticides for dinotefuran, abamectin and β-cypermethrin were 296.010 mg/L, 14.658 mg/L, and 125.908 mg/L, respectively (Table 2). The 95% confidence intervals of the LC_30_ and LC_50_ values for these insecticides were also calculated.

**Figure 4:**
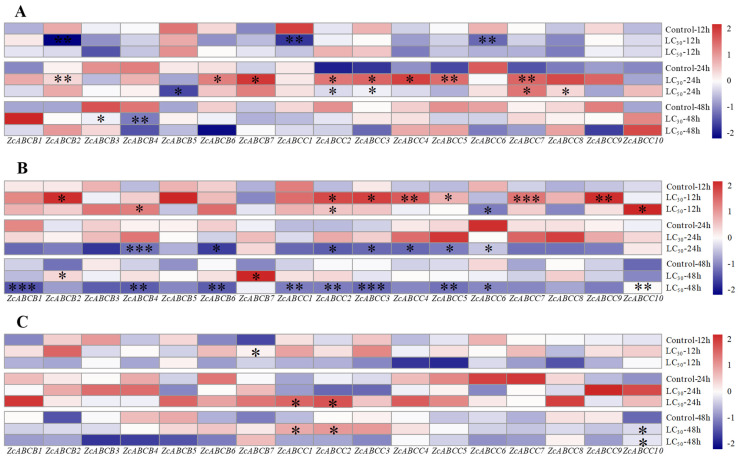
Relative expression levels of 17 ABC transporters in *Zeugodacus cucurbitae* treated with β-cypermethrin (**A**), abamectin (**B**) and dinotefuran (**C**). The relative expression of each gene challenged by three insecticides was determined by qRT-PCR. Blue indicates low expression, and red indicates high expression. Student’s *t*-test was performed for comparison with the corresponding control group, * *p* < 0.05, ** *p* < 0.01, *** *p* < 0.001.

The dosage of LC_30_ and LC_50_ insecticides were thereafter used to evaluate the induction of gene expression of ABC genes. The results showed that only *ZcABCB7* and *ZcABCC2*, among 17 selected genes that are mainly involved in detoxification metabolism, were remarkably over-expressed in *Z. cucurbitae* when challenged by a LC_30_ of these three insecticides (Figure 4). For β-cypermethrin, *ZcABCB1*, *ZcABCB6*, *ZcABCB7*, *ZcABCC2*, *ZcABCC3*, *ZcABCC4*, *ZcABCC5*, and *ZcABCC7* were significantly up-regulated 24 h after a LC_30_ dosage. *ZcABCB2*, *ZcABCC1*, and *ZcABCC6* were significantly down-regulated by the LC_30_ dosage, and *ZcABCB3* and *ZcABCB4* significantly down-regulated after 48 h (Figure 4A). After exposure to abamectin, *ZcABCB2*, *ZcABCB7*, *ZcABCC2*, *ZcABCC3*, *ZcABCC4*, *ZcABCC5*, *ZcABCC7*, and *ZcABCC9* were significantly up-regulated 12 h after dosage with the LC_30_. The mRNA expression levels were up-regulated from 1- to 2.5-fold. *ZcABCB1*, *ZcABCB4*, *ZcABCB6*, *ZcABCC1*, *ZcABCC2*, *ZcABCC3*, *ZcABCC4*, *ZcABCC5,* and *ZcABCC6* were all significantly down-regulated 24 h and 48 h after a LC_50_ dosage (Figure 4B). After a challenge with dinotefuran, only the expression of *ZcABCB7* was significantly up-regulated 12 h after a LC_30_ dosage. *ZcABCC1*, *ZcABCC2,* and *ZcABCC10* were significantly up-regulated 48 h after a LC_30_ and 24 h after LC_50_ treatments (Figure 4C).

### 3.4. Transcriptional Response of ZcABCs to β-Cypermethrin Exposure in Tissues

**Figure 5:**
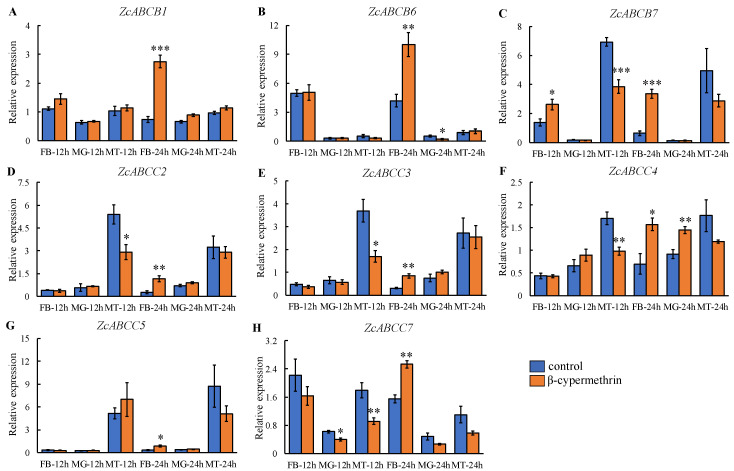
Expression of *ZcABCB1* (**A**), *ZcABCB6* (**B**), *ZcABCB7* (**C**), *ZcABCC2* (**D**), *ZcABCC3* (**E**), *ZcABCC4* (**F**), *ZcABCC5* (**G**) and *ZcABCC7* (**H**) in three tissues of *Zeugodacus cucurbitae* at 12 h and 24 h after exposure to a LC_30_ of β-cypermethrin. Student’s *t*-tests were performed for comparison with the corresponding control group, * *p* < 0.05, ** *p* < 0.01, *** *p* < 0.001.

Expression levels of *ZcABCB1*, *ZcABCB6*, *ZcABCB7*, *ZcABCC2*, *ZcABCC3*, *ZcABCC4*, *ZcABCC5*, and *ZcABCC7* within several tissues important for detoxification (midgut, fat body, and Malpighian tubules) were quantified at 12 h and 24 h after a LC_30_ exposure to β-cypermethrin. All of these ABC transporter genes were significantly induced within the fat body 24 h after exposure (Figure 5), especially *ZcABCB7* and *ZcABCC2* with a 4.25 and 3.30-fold change, respectively (Figure 5C,D). In addition, *ZcABCB7*, *ZcABCC2*, *ZcABCC3*, *ZcABCC4,* and *ZcABCC7* were significantly down-regulated in Malpighian tubules 12 h after exposure. The 17 genes showed no significant expression changes within the midgut, although *ZcABCB7*, *ZcABCC3*, *ZcABCC4,* and *ZcABCC7* were down-regulated markedly after both 12 h and 24 h.

## 4. Discussion

With the relatively recent improvements in sequencing technology, more genomes of arthropods have been sequenced and annotated, leading to an expansion of databases and ability to identify family genes. For example, there are 56 ABC transporter genes in *D. melanogaster* [31], 55 in *B*. *tabaci* [32], 55 in *Bombyx mori*, 73 in *Tribolium castaneum*, 41 in *Apis mellifera*, 52 in *Anopheles gambiae* [33], and 44 in *Diaphorina citri* [34]. In this study, a total of 49 ABC transporter genes were identified in *Z. cucurbitae* based upon the sequenced genome. These genes were divided into eight subfamilies (ABCA-ABCH) which varied based upon their functions and similarity of conserved domains. Compared to *D. melanogaster*, the A and C subfamily of *Z. cucurbitae* have relatively small numbers. The *Z. cucurbitae* ABCA subfamily has six FT and one incomplete ABC protein with one NBD domain. In silkworm, ABCAs consist of two FT, one HT, and only three have one NBD [35]. In *Bactrocera dorsalis*, ABCAs consist of five FT and two HT [36]. In contrast, all of the ABCAs subfamily genes were FTs in *A*. *gambia* [12].

The structural differences between the ABCA subfamily indicate diversity associated with the evolutionary process in insects. The analysis of the ABCB subfamily of *Z cucurbitae* revealed three FT and four HT members. This differs from that of *B*. *tabaci* in which all ABCBs members are HTs. Both FTs and HTs play important roles in arthropods [37]. For the ABCD, ABCE, ABCF, and ABCH subfamilies of *Z cucurbitae*, two, one, three, and three proteins were identified, respectively. The same numbers of these subfamily genes were also identified in other insects, indicative of a conserved evolution.

The ABCG genes constitute the largest subfamily, and the ABCH subfamily is similar to ABCG. ABCGs are involved in pigment precursor transporters, such as *brown*, *scarlet* and *white* genes in *D. melanogaster* [31,38]. The ABCH subfamily was first reported in *D. melanogaster* but has not been found in plant and fungi [32]. Hence, these genes may play unique roles in insects. In *Locusta migratoria* an ABCH subfamily member *LmABCH-9c* was demonstrated to be involved in the process of constructing a lipid-based barrier on the cuticle surface [39].

A comprehensive expression pattern of *ZcABC* genes could provide an insight into potential physiological functions associated with development and/or within tissues. In *Z. cucurbitae*, *ZcABCs* were expressed in the larva stage at a relatively low level but with a high expression in egg, pupa, and adult stages. This indicated that most of *ZcABCs* are involved in metamorphosis development. This is supported by a study of a knockdown of the *TcABCA-9A/9B* in *T*. *castaneum*, as this caused wing defects and elytral shortening at pupa and adult, and knockdown of *TcABCE-3A* and *TcABCF-2A* was lethal because of failed molting [40]. In *Z cucurbitae*, five *ZcABCA* genes exhibited a higher expression at male adult stage that increased gradually, which is consistent with results in *Helicoverpa armigera* [41]. 

Expression of *ZcABCBs* and *ZcABCCs* family genes were dynamic at all developmental stages of *Z cucurbitae*. The expression patterns were different among these genes, indicating that ABCB and ABCC subfamilies probably have minor roles during insect metamorphosis. ABCB and ABCC subfamilies are also thought to be involved in pesticide resistance and other chemicals in insects. *ZcABCE1*, *ZcABCF2*, and *ZcABCF3* were highly expressed at each stage. Hence, the expression patterns of these three genes are consistent with basic functions of biogenesis and translation regulation of ribosomes [42]. *ZcABCGs* and *ZcABCHs* were expressed in a complex fashion at all developmental stages, because of their functional diversity.

The expressions of *ZcABCs* were diverse within the different tissues. Multiple *ZcABCBs* and *ZcABCCs* were highly expressed in the Malpighian tubule and midgut, such as *ZcABCC* (1–6, 8), *ZcABCB* (2, 3, 5, 7), and *ZcABCG* (3, 5, 9, 11, 16). Furthermore, the midgut and the Malpighian tubule are a digestive organ and excretory organ, respectively, and are involved in detoxification metabolism. The expression patterns of these genes are similar to most of those from metabolic detoxification families, such as P450, glutathione *S*-transferase, and carboxylesterase [1]. For example, a knockout of three ABCC subfamily genes (*Mdr49*, *Mdr50*, and *Mdr65*) in *D. melanogaster* [43], and *Mdr49*, *Mdr50*, and *Mdr65* resulted in an increased susceptibility to insecticide. In *P*. *xylostella*, it has been demonstrated that the *pxABCC2* and *pxABCC3* proteins also functioned as receptors for BtCry1 toxins within the midgut [44]. These comparable studies provide insights into the potential function of these highly expressed ABC genes within the midgut and Malpighian tubule. The expression of the *ZcABC* gene within the fat body suggested its potential function in the transport of cuticular lipids. In *T. castaneum*, an ABC transporter gene plays an important role in cuticle lipid transport to maintain osmotic balance [40]. In this study, some *ZcABCs* were highly expressed within reproductive tissues. Furthermore, these genes were also expressed in increasing levels as the adult developed. The presence and expression of these genes within testis or ovary indicates that they could play a role in spermatic or ovarian development, respectively.

In addition to the transport of insecticides and allelochemicals, ABC transporters have also been studied as insecticide targets. ABC transporters were involved in the degradation of plant secondary metabolites in the cotton bollworm, *H*. *armigera* [5]. ABCBs are often positively correlated with field population resistance and ABCB FTs have been associated with insecticide transport and/or resistance in insects [4]. The ABCC subfamily known as MRPs, transports a range of substrates, such as drugs, endogenous compounds and glutathione, and cyclic nucleotides [16]. In *Anopheles arabiensis* and *D. melanogaster*, *ABCB* and *ABCC* genes were overexpressed in a dichlorodiphenyltrichloroethane (DDT)-resistant strain [45,46]. Hence, B and C subfamilies were selected for further analysis of the transcriptional response to the insecticides (dinotefuran, abamectin and β-cypermethrin) in this study. An analysis of the expression of the 17 candidate ABC genes was undertaken after insecticide exposure. The results showed that some of *ZcABCBs* and *ZcABCCs* were highly expressed after treatment with the insecticides. This positive correlation between induction of the ABC subfamily and insecticide resistance has also been demonstrated in other insects [16]. After insecticide treatment, the ABC gene showed up-regulation in several insects [1]. For example, a CRISPR-Cas9 knockout for *Mdr65* was more susceptible to all neuroactive insecticides tested [43]. When imidacloprid was used to treat *B. tabaci* adults, the expression of *ABCG3* was increased, and a knockdown of *ABCG3* significantly increased the mortality of adults exposed to imidacloprid [13]. In *B. dorsalis*, after insecticides exposure, more than 10 ABC genes were significantly overexpressed [36]. Two ABC transporters, *ZcABCB7* and *ZcABCC2,* were significantly up-regulated in *Z. cucurbitae* after exposure to a LC_30_ of three insecticides.

To assess xenobiotic responses, three insecticides were selected, namely, abamectin, β-cypermethrin and dinotefuran. We found that most of the *ZcABCB* and *ZcABCC* subfamily genes showed no response to dinotefuran. This may result from that neonicotinoid insecticides are more effective to Hemiptera [16,34,47]. However, *ZcABCB7*, *ZcABCC2*, *ZcABCC3*, *ZcABCC4*, *ZcABCC5,* and *ZcABCC7* were all significantly up-regulated by abamectin andβ-cypermethrin exposure. Similar results were found in *B. dorsalis* [36]. A knockdown of *BdABCB7* significantly increased the toxicity of malathion, and increased the mortality of *B. dorsalis* [36]. After exposure to β-cypermethrin, many ABCBs and ABCCs were up-regulated at 24 h, a time point consistent with in other literatures. However, after exposure to abamectin, most of these genes were up-regulated at 12 h, rather than 24 h. Furthermore, the majority of ABCBs and ABCCs had no response to dinotefuran exposure. Therefore, we used β-cypermethrin to test which tissues might play a role in detoxification metabolism After treatment of *Z cucurbitae* adults with a LC_30_ of β-cypermethrin, the midgut, Malpighian tubule, and fat body were dissected 12 h and 24 h after insecticide exposure. The genes: *ZcABCB1*, *ZcABCB6*, *ZcABCB7*, *ZcABCC2*, *ZcABCC3*, *ZcABCC4*, *ZcABCC5,* and *ZcABCC7* were specifically induced within the fat body 24 h after exposure. This result is consistent with the expression pattern of adults after β-cypermethrin exposure, indicating a detoxification related role of these genes in *Z. cucurbitae*. However, further research is required to fully elucidate this response and its mechanism of action.

Among the seven ABCB transporters identified in *Z. cucurbitae*, three of them were FTs, and could be participating in detoxification metabolism. In *D. melanogaster*, three genes (*mdr49*, *mdr50*, and *mdr65*) are part of the ABCB transporters, and disruption of the *mdr49* can increase resistance to colchicine [48]. Furthermore, knockout of these three genes renders flies more susceptible to nitenpyram and imidacloprid [43]. A number of invesigations have suggested that these gene products are involved in the transport of these chemicals [49]. In this study, *ZcABCB7*, which is homologous with *mdr49*, was mainly expressed in the midgut, fat body and Malpighian tube. The upregulation of *ZcABCB7* in *Z. cucurbitae* by three insecticides exposure indicated an insecticide metabolism related role in *Z. cucurbitae*. 

In addition to the participation of ABCB transporters in insecticide detoxification, ABCC transporters were also found to be involved in insecticide metabolism. A total of 10 *ZcABCCs* were identified in *Z. cucurbitae*. They are FTs and may have various functions in ion transport, cell-surface receptor activity and substrate translocation [50]. In humans, the ABCCs mainly comprise MRPs [3]. In *Manduca sexta*, ABCC transporters are involved in multidrug resistance and detoxification [51]. In *Chilo suppressalis*, *CsABCC8* was significantly up-regulated after chlorantraniliprole exposure and *CsABCC8* was homologous with the sulfonylurea receptor (Sur) that may participate in insecticide metabolism [52]. In this study, *ZcABCC2* and *ZcABCC3* were mainly expressed in adults and within the Malpighian tube. The significant up-regulation of these genes after exposure to three insecticides indicate that they may be involved in insecticide metabolism in *Z. cucurbitae*.

## 5. Conclusions

In summary, herein, a total of 49 ABC transporter genes were identified from *Z. cucurbitae*. All of these genes had complete ORFs and fitted to a genome scaffold. The phylogenetic relation and subfamily classifications were determined based on the presence of clear homologous orthologs in *D. melanogaster*. A bioassay that assessed responses to three kinds of insecticides was performed in *Z. cucurbitae*, and the LC_50_ dosage of β-cypermethrin, abamectin and dinotefuran quantified as 125.908, 14.658, and 296.010 mg/mL, respectively. Two prominent subfamily members, ABCB and ABCC, were selected for the evaluation of these three insecticides, with results that indicated that *ZcABCB7* and *ZcABCC2* may be associated with insecticide tolerance. Dissected tissues from flies exposed to β-cypermethrin, revealed that the *ZcABCB1*, *ZcABCB6*, *ZcABCB7*, *ZcABCC2*, *ZcABCC3*, *ZcABCC4*, *ZcABCC5*, and *ZcABCC7* genes were significantly expressed in the fat body, implying that the fat body may be the major tissue to transport pyrethroids.

## Figures and Tables

**Table 1:** Characterization of 49 ABC transporter proteins in *Zeugodacus cucurbitae*.

Subfamily	Gene	Gene IDc	Length (AA)	Topology ^a^	N-Glc ^b^	O-Glc ^c^
A	*ZcABCA1*	LOC105218935	1768	(5/7TMD-NBD)_2_	14	13
A	*ZcABCA2*	LOC105214217	1210	(6TMD-NBD)_2_	8	1
A	*ZcABCA3*	LOC105214171	458	NBD	1	1
A	*ZcABCA4*	LOC105214218	1685	(7/6TMD-NBD)_2_	11	6
A	*ZcABCA5*	LOC105210581	1610	(7/6TMD-NBD)_2_	3	15
A	*ZcABCA6*	LOC105219391	1568	(6TMD-NBD)_2_	9	15
A	*ZcABCA7*	LOC105215594	1957	(6/7TMD-NBD)_2_	12	6
B	*ZcABCB1*	LOC105218240	850	11TMD-NBD	7	1
B	*ZcABCB2*	LOC105221102	719	6TMD-NBD	5	4
B	*ZcABCB3*	LOC105218701	732	3TMD-NBD	3	9
B	*ZcABCB4*	LOC105217120	673	11TMD-NBD	6	2
B	*ZcABCB5*	LOC105213990	1295	(6/5TMD-NBD)_2_	3	9
B	*ZcABCB6*	LOC105212618	1296	(7/6TMD-NBD)_2_	7	7
B	*ZcABCB7*	LOC105212186	1302	(6/5TMD-NBD)_2_	9	4
C	*ZcABCC1*	LOC105214114	1352	(6/5TMD-NBD)_2_	4	4
C	*ZcABCC2*	LOC105214112	1353	(7/5TMD-NBD)_2_	9	4
C	*ZcABCC3*	LOC105214120	1346	(7/5TMD-NBD)_2_	7	5
C	*ZcABCC4*	LOC105214115	1365	(7/5TMD-NBD)_2_	5	11
C	*ZcABCC5*	LOC105209430	1358	(4/5TMD-NBD)_2_	9	8
C	*ZcABCC6*	LOC105214620	1295	(3/4TMD-NBD)_2_	12	8
C	*ZcABCC7*	LOC105217830	1408	(6/5TMD-NBD)_2_	5	5
C	*ZcABCC8*	LOC105218206	1552	7TMD-(5TMD-NBD)_2_	7	4
C	*ZcABCC9*	LOC105210838	1494	5TMD-(6TMD-NBD)_2_	10	1
C	*ZcABCC10*	LOC105217701	1313	4TMD-NBD	15	59
D	*ZcABCD1*	LOC105218326	737	4TMD-NBD	2	2
D	*ZcABCD2*	LOC105208882	658	3TMD-NBD	4	6
E	*ZcABCE1*	LOC105221159	611	NBD-NBD	1	6
F	*ZcABCF1*	LOC105212708	897	NBD-NBD	8	22
F	*ZcABCF2*	LOC105216261	613	NBD-NBD	4	8
F	*ZcABCF3*	LOC105208450	708	NBD-NBD	4	8
G	*ZcABCG1*	LOC105220708	607	NBD-7TMD	3	9
G	*ZcABCG2*	LOC105220707	891	NBD-7TMD	7	36
G	*ZcABCG3*	LOC105215537	679	NBD-5TMD	4	21
G	*ZcABCG4*	LOC105209688	1124	NBD-5TMD	9	85
G	*ZcABCG5*	LOC105217559	666	NBD-7TMD	2	7
G	*ZcABCG6*	LOC105215923	641	NBD-7TMD	3	12
G	*ZcABCG7*	LOC105209629	638	NBD-7TMD	6	15
G	*ZcABCG8*	LOC105209627	724	NBD-6TMD	2	0
G	*ZcABCG9*	LOC105218359	615	NBD-5TMD	4	6
G	*ZcABCG10*	LOC105213062	688	NBD-6TMD	3	0
G	*ZcABCG11*	LOC105209626	806	NBD-6TMD	5	46
G	*ZcABCG12*	LOC105217960	619	NBD-6TMD	6	6
G	*ZcABCG13*	LOC105217961	1388	NBD-5TMD-NBD-8TMD	9	26
G	*ZcABCG14*	LOC105213059	862	NBD-7TMD	7	31
G	*ZcABCG15*	LOC105213060	730	NBD-6TMD	7	2
G	*ZcABCG16*	LOC105212186	743	NBD-5TMD	4	28
H	*ZcABCH1*	LOC105217189	775	NBD-7TMD	10	15
H	*ZcABCH2*	LOC105217218	764	NBD-7TMD	10	12
H	*ZcABCH3*	LOC105218730	725	NBD-7TMD	4	9

^a^ Transmembrane helices (TMs) were predicted using the TMHMM 2.0 server. ^b^ A N-glycosylation sites were predicted using NetNGlyc 1.0 server; only N-glycosylation site with a “potential” score > 0.5 and with a jury agreement were considered. ^c^ O-glycosylation sites were predicted using NetOGlyc 4.0 server. If the G-score was higher than 0.5 the residue was considered to be O-glycosylated, the total number of O-glycosylated sites (glycosylated serine and threonine) is shown.

**Table 2:** Toxicity bioassay of three insecticides against *Zeugodacus cucurbitae*.

Insecticides	*n*	Slope(±SE)	LC_30_ (mg/L)	95% CI ^a^	LC_50_ (mg/L)	95% CI	*χ* ^2 b^
β-Cypermethrin	300	5.376 ± 0.541	100.580	91.336–109.824	125.908	115.291–138.841	0.443
Abamectin	360	2.682 ± 0.234	9.344	6.734–11.963	14.658	11.405–18.723	5.1793
Dinotefuran	331	4.045 ± 0.375	219.616	118.042–296.924	296.010	196.121–398.702	8.8061

^a^ CI means confidence interval. ^b^ *χ*^2^ value for goodness of fit test.

## Data Availability

Data is contained within the article and supplementary material.

## Supplementary Materials

The following are available online at https://www.mdpi.com/2075-4450/12/3/270/s1, Table S1. Primers for the ABC transporter genes for RT-qPCR.
